# Immune Responses to *C. trachomatis* Infections in a Non-Human Primate Model

**DOI:** 10.1155/S1064744996000348

**Published:** 1996

**Authors:** Dorothy L. Patton

**Affiliations:** 1 Department of Obstetrics and Gynecology University of Washington Seattle WA USA; 2 Box 356460 St. 1959 NE Pacific Street Bldg Health Sciences Seattle Washington 98195-6460 RR609 USA


Infectious Diseases in Obstetrics and Gynecology 4:159–162 (1996)
© 1996 Wiley-Liss, Inc.

### Immune Responses to *C. trachomatis* Infections in a Non-Human Primate Model

**Dorothy L. Patton**

*Department of Obstetrics and Gynecology, University of Washington, Seattle, WA*

KEY WORDS

*Chlamydia trachomatis;* animal model; salpingitis; cervicitis; cytokines

Chlamydial infections are the most common bacterial STD in the United States, with an estimated annual incidence of 4 million cases and costs in excess of $2 billion. Pelvic inflammatory disease (PID) (i.e., acute salpingitis) is frequently a consequence of sexually transmitted diseases, including gonococcal, mycoplasmal, and chlamydial infection, and is usually the result of progression of disease from the lower to the upper reproductive tract. Approximately 1 million women are treated for PID in the United States annually, and one-quarter of these women are affected with serious sequelae, including ectopic pregnancy and tubal infertility.[1] As many as half of these PID cases are caused by chlamydiae. Such infections are now recognized as a major public health problem. However, a limited understanding of the immuno-pathogenesis of PID still exists. This has been due in part to the limited number of clinical specimens available for study and the continued need to develop appropriate and suitable animal models in which to study *Chlamydia trachomatis* in particular.[1–4]

### PIG-TAILED MACAQUE CERVICAL INOCULATION MODEL

For the past decade, we have used pig-tailed monkeys to study the pathogenesis of chlamydial cervicitis and salpingitis. Since the normal reproductive biology of the pig-tailed monkey is well defined, we anticipated that this model would be useful for functional studies following induction of cervicitis and salpingitis and for easily recognizing abnormal morphology in infected animals. Initially, our studies were designed to determine whether the pig-tailed monkey would be an appropriate animal model in which to study the pathogenesis of chlamydial cervicitis and salpingitis. As described below, the immune responses and histopathologic characteristics of chlamydial infection in this model mimic human cervicitis and salpingitis, although only limited data in humans are available. Thus, we have continued to use the pig-tailed monkey model to evaluate *C. trachomatis* infection in the lower and upper genital tracts.

### Repeated Cervical Inoculation With *C. trachomatis* After Culture-Negative Intervals[5]

Seven pig-tailed macaques were inoculated in the cervix with a serovar D strain, while 3 control monkeys were cervically inoculated with uninfected McCoy cells only. The animals that received viable *C. trachomatis* were than reinoculated, but only after 2 to 3 consecutive weeks of culture-negative specimens. After spontaneous cessation of cervical shedding of *C. trachomatis*, repeated inoculation either failed to produce infection or resulted in infection of shorter duration with lower inclusion counts. Thus, the initial cervical infection persisted for 1–15 weeks (average, 9.3 weeks), while reinoculation of the cervix failed in 3 of 7 animals, and among the 4 animals who were infected, persisted only 4.8 weeks on average. Among 2 animals infected a third time, the third infection averaged 1.5 weeks. The highest chlamydial inclusion count in primary infec-

Address correspondence Box 356460, St. 1959 NE Pacific Street, Bldg Health Sciences, 98195-6460 RR609, Seattle, Washington.

Received September 15, 1996
Accepted October 1, 1996

Article tions was not reached until after 5 weeks in 3 animals, and after 12–13 weeks in the other 3. In 1 animal the culture was positive on one occasion only. In contrast, the maximum inclusion count after the second inoculation was much reduced as compared with those after initial infection. Laparoscopy after the initial infection showed uterine erythema in 1 of 4 animals and in all animals after the second cervical inoculation with *C. trachomatis.* Tubal edema was also a frequent observation, but tubal cultures were consistently negative. No peritubal or periadnexal adhesions were observed during this experiment. In conclusion, after repeated cervical inoculations given when cervical shedding had ceased prior to rechallenge, either no infection developed or an infection of shorter duration was produced, suggesting that partially protective immunity occurred at the level of the cervix. Induction of tubal scarring or adhesions was not observed.

### Acute Salpingitis Induced by Repeated Weekly Cervical Inoculations of *C. trachomatis*[6]

Because upper tract disease was not observed following repeated cervical inoculations, we increased the frequency of the cervical inoculations to once weekly for 5 weeks. A serovar D strain was used and 3 monkeys were studied. Laparoscopies and videomicroscopy were performed at weekly intervals to assess gross morphology and the presence of *C. tarchomatis* by culture of the cervix and fallopian tubes. After initial inoculation, chlamydia was reisolated from the cervix in all 3 monkeys and uterine erythema occurred in 2 of 3 monkeys. After 3 weeks of cervical inoculations, mild omental and peritubal adhesions developed in 1 monkey and severe peritubal and omental adhesions developed in another monkey. In this latter monkey, chlamydia was isolated from the fallopian tube at the time of the fourth laparoscopy. Plasma cell cervicitis, endometritis and salpingitis were also documented in this particular monkey. We conclude from these experiments that upward migration of chlamydia from the cervix into the fallopian tube occurred and produced acute salpingitis similar to that observed in women.

### Repeated Cervical Infection Followed by a Single Tubal Challenge with *C. trachomatis*[7]

In a subsequent series of experiments, we tested the hypothesis that more severe tubal disease could be induced if the animal first received repeated cervical inoculations, followed by direct inoculation of the fallopian tube. Eleven pig-tailed macaques were cervically inoculated between 2 and 5 times with *C. trachomatis* serovars D and F. Fallopian tubes were then inoculated with serovar D or F 1 week after the last cervical inoculation. In addition, three control monkeys received 1 direct tubal inoculation without previous cervical inoculation. The production of cervical infection was confirmed by isolating *C. trachomatis* from the endocervix in 13 of 14 monkeys. Chlamydia was isolated from the endosalpinx in four animals, but only after the tubal inoculation. Antibody to *C. trachomatis* was detected in post-infection sera of all 14 animals. Tubal edema occurred in 7 of 11 animals after the first cervical inoculation, and uterine erythema occurred in all 11 after the second cervical inoculation. Before tubal inoculation, peritubal adhesions were present in 0 of 7 monkeys given 3 or fewer cervical inoculations vs. all 4 given 5 cervical inoculations ($p < .01$). After direct tubal inoculation, however, peritubal adhesions became more prominent. Hysterectomy specimens were obtained from 11 animals, of which 9 had plasma cell endometritis and 9 had salpingitis. Two control monkeys developed minor adhesions, while the other had none. One fallopian tube in 2 of the 3 controls showed mild plasma cell infiltrates, but no evidence of endometritis was observed in the controls. The histopathologic findings in these monkeys were characteristic of chlamydial cervicitis, endometritis, and salpingitis in humans.

In summary, the major findings of interest in this study included: 1) frequent production of uterine erythema, tubal edema, and peritubal adhesions following repeated cervical infection; and 2) production of endometritis and endosalpingitis following tubal infection, with or without prior cervical infection. In the absence of direct tubal inoculation, development of peritubal adhesions required 5 weekly cervical inoculations.

### Mediators of Inflammation Associated With Scarring and Fibrosis[8]

The spectrum of cytokines expressed during persistent infection with *C. trachomatis* has not been described. Studies to determine the cytokine mRNA profile in infected target tissues were carried out to help characterize the local immune response occurring in chronically-infected fallopian tubes, the

TABLE 1. Cytokine mRNA in tubes detected by RT-PCR in 6 week *C. trachomatis*-infected macaques (n = 2) and in uninfected macaques (n = 3)*

| | Infected #1 | Infected #2 | Uninfected #1 | Uninfected #2 | Uninfected #3 |
|---|---|---|---|---|---|
| IFN-γ | 3+ | 3+ | – | +/– | – |
| IL-4 | – | – | – | – | – |
| IL-5 | +/– | +/– | – | +/– | +/– |
| IL-6 | 3+ | 3+ | – | +/– | +/– |
| IL-10 | 1+ | 2+ | – | – | – |
| PDGF-β | 2+ | 2+ | – | – | – |
| TGF-β | 3+ | 4+ | – | +/– | 1+ |

*Each sample was semi-quantitatively scored for the PCR product for each cytokine. Samples scored as 3+ had the most cytokine PCR product, 2+ and 1+ successively less quantitites of PCR product, while +/– mean the PCR product was barely detectable, and – meant the PCR product was absent.

tissue most damaged by *C. trachomatis* infection. The cytokines expressed in *C. trachomatis*-infected tissues were detected using reverse transcriptase combined with polymerase chain reaction (RT-PCR). A series of oligonucleotides were developed that detected IFN-γ, IL-4, IL-5, IL-6, IL-10, PDGF-β and TGF-β mRNA by RT-PCR from RNA extracted from mitogen-stimulated macaque peripheral blood mononuclear cells. The quantity of cDNA in each tissue was normalized for constitutive mRNA for hypoxanthine phosphoribosyl transferase using quantitative competitive PCR.[8] Semi-quantitative PCR was used to determine the relative quantity of selected cytokine mRNA. Macaque fallopian tubes infected for 6 and 12 week durations were studied. The 12 week infected tubes were found to have no increase in cytokine mRNA compared with uninfected controls (data not shown). This correlated with histologic findings of end stage fibrosis and scarring with relatively few inflammatory cells present. It seems likely that the synthesis of cytokines related to the production of fibrosis and scarring preceded the 12 week end point. This hypothesis was supported by detection of cytokine mRNA for IFN-γ, IL-6, Il-10, PDGF-β and TGF-β, but not IL-4 and IL-5, in increased quantities in the 6 week infected tubes vs. unifected tubes (Table 1). This correlated with a much more active cellular infiltration in the 6 week than in the 12 week infected tubes, including the presence of numerous lymphocytes and macrophages. The finding of IFN-γ production in the absence of detectable IL-4 and IL-5 production suggested that Th1 type cytokines dominate the chronic immune response to *C. trachomatis* infection in tubes.[9] Though Th1 responses generally help control intracellular pathogens, exaggeration of Th1 responses due to a persistent pathogen may lead to scarring and fibrosis.

The presence of PDGF-β and TGF-β mRNA in 6 week infected tubes suggests that these mediators may be involved in the generation of tubal fibrosis and scarring. Both PDGF-β and TGF-β are generally derived from macrophages during an inflammatory response.[10,11] Both of these cytokines have been implicated in processes leading to fibrosis and interference with TGF-β function has been shown to prevent scarring in a glomerulonephritis model.[10–12]

Studies have been initiated to confirm these results using cytokine detection assays. IFN-γ was detected using ELISA in the peritoneal fluid of 78 of 11 chronically-infected macaques but in none of 2 uninfected control macaques (Table 2). IL-6 was detected using the B9 bioassay[13] in peritoneal fluid of 6 of 8 chronically infected animals, but in 0 of 2 uninfected controls. IL-10 was detected by ELISA in peritoneal fluids of 7 of 11 infected animals but in neither of the 2 uninfected macaques. These results confirm some of the findings from the RT-PCR assay of cytokine mRNA. Since both IL-6 and IL-10 are produced by both Th1 and Th2 $CD4^+$ T cells in humans,[14] both cytokines could be produced by both types of $CD4^+$ T cells in macaques. Since IL-4 and IL-5 mRNA could not be detected by RT-PCR, it seems likely that the IFN-γ, IL-6, and IL-10 are produced by a predominantly Th1 immune response, though further studies are necessary to confirm this hypothesis. The high levels of IL-10 found are of interest, because IL-10 has been shown to antagonize the killing of intracellular pathogens by IFN-γ activated macrophages. Thus, high levels of IL-10 may be associated with persistence of *C. trachomatis*.

TABLE 2. Presence of cytokines in peritoneal fluids of infected and uninfected macaques*

| | IFN-γ (IU/ml) | | | IL-6 (pg/ml) | | | IL-10 (pg/ml) | | |
|---|---|---|---|---|---|---|---|---|---|
| | +/total | range | mean | +/total | range | mean | +/total | range | mean |
| Ct inf | 7/11 macaques | <2 – >50 | >20 | 6/8 macaques | <10–420 | 157 | 7/10 macaques | <200–14,000 | 3966 |
| No Ct | 0/2 macaques | <2 | <2 | 0/2 macaques | <10 | <10 | 0/2 macaques | <200 | <200 |

*Ct-inf = peritoneal fluid from *C. trachomatis* infected macaques taken at 2 or 4 weeks after infection; No Ct = peritoneal fluid from uninfected macaques; +/total = the number of samples that exceed the lower detection limit for the cytokine/the number of samples tested.

### SUMMARY

Our understanding of the pathogenesis of chlamydial PID/salpingitis and its sequelae is minimal. Current knowledge of the pathogenesis has been derived primarily from nonhuman primate and small animal experimental models because comprehensive data and reproductive tract tissues from women are limited. We have utilized the pig-tailed macaque in situ model to study the natural history and mechanisms of pathogenesis of chlamydial lower and upper genital tract infections. More recently, the direction of our research has focused on the immune response and immunopathology associated with *C. trachomatis* tubal disease. Continued studies will advance our understanding of scarring and fibrosis associated with human genital tract chlamydial infections and may lead to the prevention of its sequelae, tubal infertility and ectopic pregnancy.

### REFERENCES

1. Centers for Disease Control. 1990 Division of STD/HIV prevention annual report. U.S. Department of Health and Human Services, Public Health Service, 1990.
2. Quinn TC, Cates W Jr.: The epidemiology of sexually transmitted diseases, 1990. In: Quinn TC, Gallin JI, Fauci AS (eds): Advances in Host Defense Mechanisms, Vol 8: Immunopathogenesis of Sexually Transmitted Diseases. New York: Raven Press, 1991.
3. Washington AE, Johnson RE, Sanders LL, et al.: Incidence of *Chlamydia trachomatis* infections in the United States: Using reported *Neisseria gonorrhoeae* as a surrogate. In: Oriel D, Ridgeway G, Schachter J, et al. (eds): Chlamydia Infections. Cambridge, England: Cambridge University Press, 1986;487.
4. Washington AE, Johnson RE, Sanders LL: *Chlamydia trachomatis* infections in the United States: What are they costing us? JAMA 257:2070–2072, 1987.
5. Wolner-Hanssen P, Patton DL, Holmes KK: Protective immunity in pig-tailed macaques after cervical infection with *Chlamydia trachomatis*. Sex Trans Dis 18:21–25, 1991.
6. Patton DL, Wolner-Hanssen P, Cosgrove SJ, Thompson JL, Holmes KK: Acute salpingitis in a pig-tailed macaque induced by cervical inoculations of *Chlamydia trachomatis*. Abstr. No. 20. Presented at Infectious Disease Society of Obstetrics and Gynecology, Seattle WA, August 7–11, 1990.
7. Patton DL, Wolner-Hanssen P, Holmes KK: The effects of *Chlamydia trachomatis* on the female reproductive tract of the *Macaca nemestrina* after a single tubal challenge following repeated cervical inoculations. Obstet Gynecol 76:643–650, 1990.
8. Van Voorhis WC, Barrett LK, Cosgrove Sweeney YT, Kuo CC, Patton DL: Analysis of lymphocyte phenotype and cytokine activity in the inflammatory infiltrates of the upper genital tract of female macaques infected with *Chlamydia trachomatis*. J Infect Dis 174:647–50, 1996.
9. Mosmann TR, Coffman RL: $Th_1$ and $Th_2$ cells: Different patterns of lymphokine secretion lead to different functional properties. Ann Rev Immun 7:145–73, 1989.
10. Shaw RJ, Benedict SH, Clark RAF, King Jr, TE: Pathogenesis of pulmonary fibrosis in interstitial lung disease: Alveolar macrophage PDGF(β) gene activation and up-regulation by interferon gamma. Am Rev Resp Dis 143:167–173, 1991.
11. Border WA, Ruoslahti E: Transforming growth factor-beta in disease: The dark side of tissue repair. J Clin Invest 90:1–7, 1992.
12. Border WA, Noble NA, Yamamoto T, Harper JR, Yamaguchi Y, Pierschbacher MD, Ruoslahti E: Natural inhibitor of transforming growth factor-β protects against protects against the scarring in experimental kidney disease. Nature 360:361–364, 1992.
13. Brakenhoff PJ. De Groot ER, Evers RF, Pannekoek H, Aarden LA: Molecular cloning and expression of hybridoma growth factor in *Escherichia coli*. J Immunol 139:4116–21, 1987.
14. Del Prete G, De Carli M, Almerigogna F, Guidizi MG, Biagiotti R, Romagnani S: Human IL-10 is produced by both type 1 helper ($Th_1$) and type 2 helper ($Th_2$) T cell clones and inhibits their antigen-specific proliferation and cytokine production. J Immunol 150:353–360, 1993.
